# Case Report: Hemophagocytic Lymphohistiocytosis Prior to the Onset of Leukemia in a Boy With *CDK13*-Related Disorder

**DOI:** 10.3389/fgene.2022.858668

**Published:** 2022-05-16

**Authors:** Dongyan Cui, Songmi Wang, Ai Zhang, Aiguo Liu, Qun Hu

**Affiliations:** Department of Pediatric Hematology and Oncology, Tongji Hospital, Tongji Medical College, Huazhong University of Science and Technology, Wuhan, China

**Keywords:** CDK13-related disorder, HLH hemophagocytic lymphohistiocytosis, acute lymphobastic leukemia, craniofacial dysmorphic features, children

## Abstract

Cardinal features of *CDK13*-related disorders are characterized by intellectual disability, developmental delay, dysmorphic facial features, structural heart defect and structural brain abnormality. A 9-year-old boy presented with intellectual disability, development delay, characteristic craniofacial features, brain malformation, cryptorchidism, autism spectrum disorder, and recently, recurrent hemophagocytic lymphohistiocytosis (HLH) in a half year period. Further investigation revealed the diagnosis of *CDK13*-related disorder. Finally, we found the underlying cause of HLH is acute lymphoblastic leukemia. Probably leukemia was a coincidental finding in this boy with *CDK13-*related disorder, but the case herein suggests that individuals with *CDK13*-related disorder also face risk of developing cancers. Further detailed information could enable us to clarify this presentation because of only limited investigation in affected cases.

## Introduction

The protein encoded by cyclin-dependent kinase 13 (*CDK13*) gene is a member of the cyclin-dependent serine/threonine protein kinase family. Members of this family are well known for their essential roles as master switches in cell cycle control (Even et al., 2016). *CDK12* and *CDK13* both are heterodimers containing a Cyclin K subunit, and they are structurally similar kinases. Many studies on *CDK12* have clearly shown that it is a tumor suppressor, notably for ovarian and prostate cancers (Greenleaf, 2019; Fan et al., 2020). In contrast, the exact function of *CDK13* has not yet been determined. Recent studies have linked heterozygous variants in *CDK13* to a syndromic form of intellectual disability and other abnormalities with variable phenotypes (Sifrim et al., 2016; Hamilton et al., 2018; van den Akker et al., 2018). Cardinal features of *CDK13*-related disorders are characterized by intellectual disability, developmental delay, dysmorphic facial features, feeding difficulties, structural heart defect and structural brain abnormality. Additional features are widely described with multisystem involvement (Hamilton and Suri, 2019). However, no malignant diseases have been reported in those with *CDK13*-related disorder. Herein, we report a rare case of a boy with *CDK13*-related disorder, who presented with recurrent hemophagocytic lymphohistiocytosis (HLH) initially with progression to leukemia in a half year period.

## Case Presentation

This boy was the first child of non-consanguineous Chinese parents and born at term with normal physical examination apart from mild hypotonia. During the first years of life, he presented with obviously delayed developmental milestones: he gained head control at 8 months, and walked alone without support at 3.5 years of age. Feeding difficulties were not reported but he had a chronic constipation. He had a history of febrile convulsions in infancy. He was diagnosed with cerebral hypoplasia at 8 months, cryptorchidism at 1 year, and autism spectrum disorder (ASD) at 5 years of age. His family history was unremarkable.

At the age of 9 years, this boy presented with recurrent fever of unknown origin and experienced a prolonged hospitalization in the local hospital from April to May 2019. Other symptoms/signs included anorexia, low mood, obvious hypotonia, increased sleep and reduced performance over usual. Subjective symptoms of discomfort were limited due to his communication deficits. His laboratory evaluation revealed an excessive inflammation and multiorgan involvement with clinically mild respiratory infections but without evidence of malignancies as summarized in Table 1 (first, 02/04 to 08/05, 2019). He received intravenous immunoglobulin (IVIG) and anti-infective therapy with third generation cephalosporins but also higher classes of antibiotics for several weeks, but his symptoms did not improve until receiving a short course of corticosteroid. He was discharged in a stable condition with a clinical diagnosis of a suspicion of HLH.

**TABLE 1:** Clinical features and laboratory parameters of the patient during attack period of HLH.

Clinical Finding	1st	2nd	3rd
	02/04 to 08/05, 2019	06/09 to 30/09, 2019	23/10 to 23/11, 2019
Fever >38.5 °C	Yes	Yes	Yes
Splenomegaly	No	No	No
Serum ferritin, µg/L (RI: 30–400)	>1,500	9,467.7	7,527.9
Triglycerides, µmol/L (RI: < 1.70)	1.58	2.56	3.69	
	
Fibrinogen, g/L (RI: 1.80–4.00)	3.53	1.67	2.94
Soluble CD25, U/mL (RI: 223–710)	624	984	1,142
NK cell activity (RI: ≥15.11%)	4.37%	ND	3.30%
Bone marrow evaluation	Hemophagocytosis	ND	ALL
CNS involvement	Anorexia, hypotonia, and increased sleep	Anorexia, hypotonia, and increased sleep	Anorexia, hypotonia, and increased sleep
Complete blood count			
ANC, ×10^9^/L (RI: 1.50–8.50)	0.98	0.56	2.18
Hb, g/L (RI: 120–160)	96	110	103
PLT, ×10^9^/L (RI: 150–450)	142	61	174
Infection related parameters			
CRP, mg/L (RI: < 0.5)	19.8	35.0	5.9
Concurrent infections	URTI	URTI	Pneumonia
Microbiology^a^	No	No	No
Hepatic function			
ALT, U/L (RI: < 40)	180	525	169
AST, U/L (RI: <40)	228	370	468
LDH, U/L (RI: 120–300)	684	1,150	1,122
Total bilirubin, µmol/L (RI: ≤ 26)	7.8	5.9	10.1
Immunologic testing			
NK cell function/degranulation	ND	ND	Normal
NK cell surface expression of granzyme B proteins	ND	ND	Reduction
Immunoglobulin levels	ND	ND	Low level of IgM, normal level of IgA and IgG
Lymphocyte subsets	ND	ND	Low level of NK cell
Other evaluation			
Echocardiogram	Normal	Normal	Normal
Chest CT	Normal	Normal	Pneumonia
Brain MRI scan	Cerebral dysplasia	ND	Cerebral atrophy
CSF analysis	Normal	Normal	ND
PET/CT	No malignancies	ND	ND
Genetic testing	ND	*CDK13*-related disorder	ND
Treatment and outcomes			
Treatment	IVIG + corticosteroid	Corticosteroid	Corticosteroid + CsA + VP16
Outcomes	Corticosteroid-free clinical remission for 4 months	Clinical remission with corticosteroid but relapsed after tapering	Clinical remission with chemotherapy but died after treatment discontinuation

Abbreviationsare as follows: ND, not determined; RI, reference intervals; CNS, central nervous system; ANC, absolute neutrophil count; Hb, hemoglobin; PLT, platelet count; CSF, cerebrospinal fluid; URTI, upper respiratory tract infection; ALL, acute lymphoblastic leukemia; IVIG, intravenous immunoglobulin; CsA, cyclosporine A; VP16, etoposide.

a This result includes cultures of potentially infected body fluids, viral testing for EB, virus, cytomegalovirus, adenovirus and other suspected viruses.

However unfortunately, he suffered a relapse within 4 months. At the second admission in the local hospital, his laboratory evaluation strongly resembled previous records, as shown in Table 1 (second, 06/09 to 30/09, 2019). Treatment with corticosteroid was initiated and his condition improved significantly in the short term. Considering his congenital anomalies, trio-based whole exome sequencing (WES) was performed using peripheral blood samples from this patient and his parents.

**FIGURE 1:**
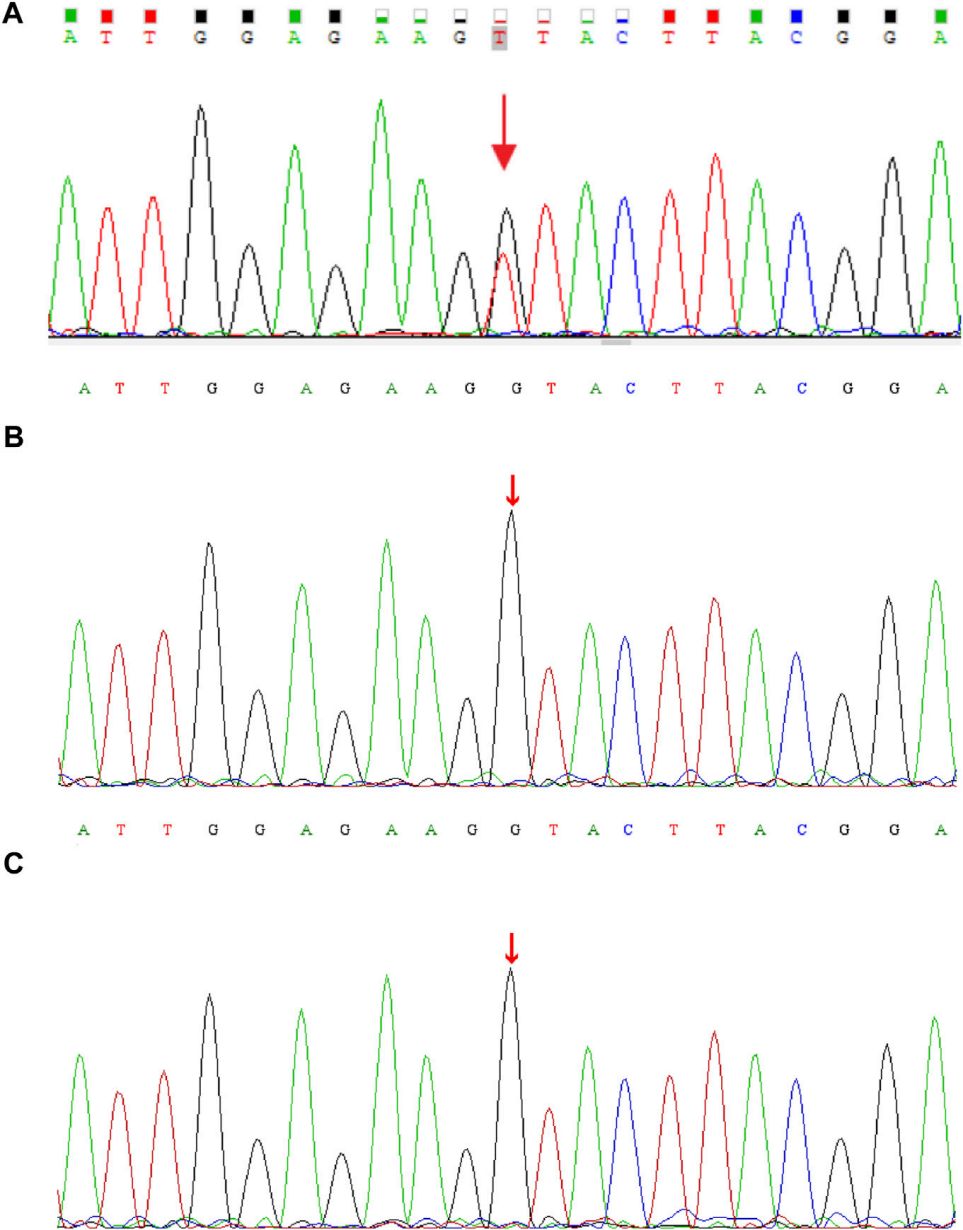
DNA electrophoregram with the (C)2141G > T [p.G714 V] in CDK13 gene of **(A)** proband **(B)** father, and **(C)** mother.

**FIGURE 2:**
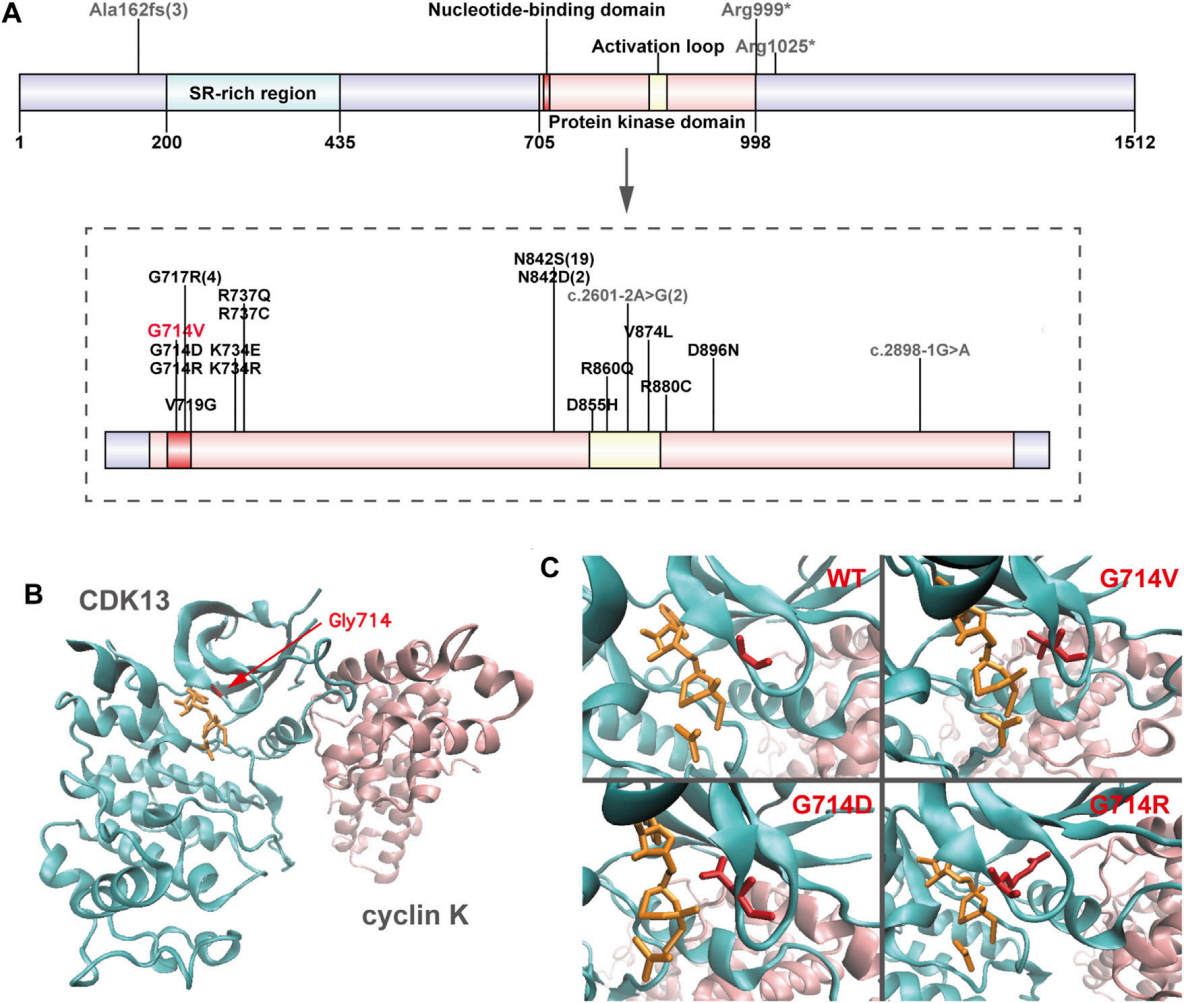
Overview of CDK13 variants **(A)** Location of pathogenic variants in the CDK13 protein of all presently reported individuals. Missense variants are annotated in black (presently reported variant in red), and others in grey. Numbers in brackets refer to the corresponding variants presented in more than one individual. Up to half (21/46) of the variants perturb the wild-type asparagine residue at amino acid position 842. **(B)** Crystal structure of one heterodimer (chains A-B) of CDK13-cyclin K (PDB id: 5efq); CDK13 and cyclin K are shown in new-cartoon format, colored cyan and pink, respectively, with sidechains of selected residues (red) and ligands (orange) shown in licorice format. **(C)** The exact positions of CDK13 residue 714 in wild-type (WT) CDK13, Gly714Val (G714 V) variant, Gly714Asp (G714D) variant, and Gly714Arg (G714R) variant, showing in red licorice format.

Genomic DNA was extracted from each sample, to an average size of 180 bp with a Bioruptor sonicator (Diagenode). Paired-end sequencing libraries then were prepared using a DNA sample prep reagent set 1 (NEBNext). Library preparation included end repair, adapter ligation and PCR enrichment, and was carried out as recommended by Illumina protocols. All sequencing was performed on the Nova seq (6,000 platform (Illumina) (MyGenostics Inc. Beijing, China). Variants were further annotated by ANNOVAR (Wang et al., 2010) and associated with multiple databases, such as, 1000genomes, ESP6500, dbSNP, ExAC, Inhouse (MyGenostics), ClinVar, and the Human Gene Mutation Database (HGMD). Variants with a minor allele frequency of less than 5% in population databases or were presented in variant databases were included in the analysis. Filtered candidate variants were confirmed by Sanger sequencing. The American College of Medical Genetics and Genomics (ACMG) Standard guidelines for the interpretation of sequence variants were followed in this study. A heterozygous missense variant in *CDK13* gene located on 7p14.1, NM_003,718, exon 4, c.2141G > T (p.G714V) was identified (full report of the variant found by WES in the trio was summarized in Supplementary Table S1), and Sanger sequencing showed the variant was *de novo* since his unaffected parents did not have the variant (Figure 1). Residue Gly714, lying within the nucleotide binding domain of *CDK13* (Figure 2), is highly conserved, and this variant is predicted to be pathogenic by SIFT, PolyPhen-2, MutationTaster, GERP++ and REVEL. This variant is not found in all population databases, and has not been described in variant databases and therefore is novel (Figure 2A), and moreover, alternative variants in the same position are classified as pathogenic by ClinVar. This novel sidechain is putative to cause changes in the *CDK13* structure (Figures 2B,C). In summary, c.2141G > T (p.G714 V) is considered to be likely pathogenic according to ACMG (PS2+PM2+PP3). Therefore, the diagnosis of *CDK13*-related disorder is made.

**FIGURE 3:**
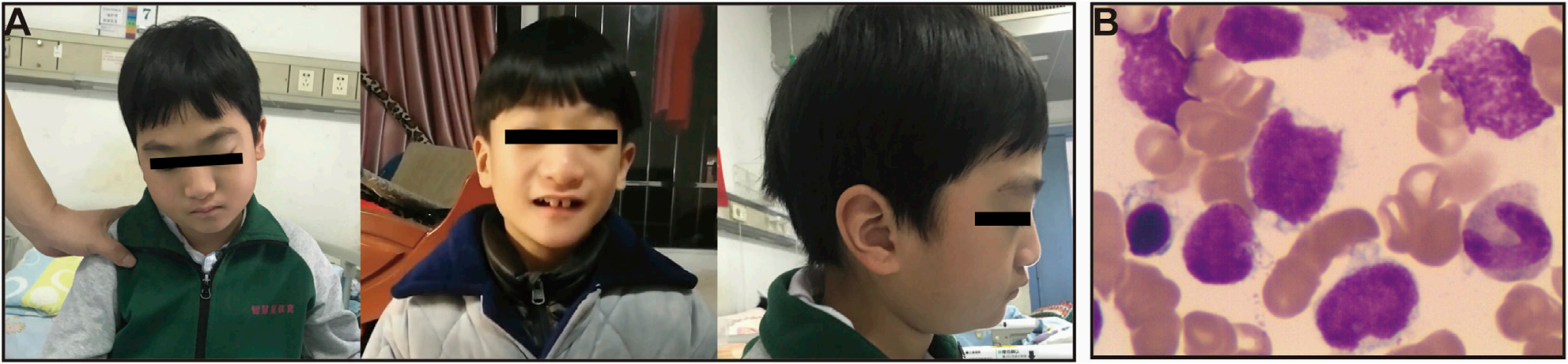
Facial features of the patient with CDK13-related disorder and bone marrow smears. **(A)** Anterior and profile facial photographs show his facial anomalies with mild exophthalmos, blepharoptosis, broad nasal bridge, big front teeth with wide space, low set and posteriorly rotated ears. He presented with drowsiness, emotional apathy after HLH onset (Left). **(B)** Bone marrow smears showing morphologic blasts cells with a proportion of 48% at diagnosis of acute lymphoblastic leukemia (ALL).

At the end of October 2019, he suffered a second relapse in the course of tapering corticosteroid, and finally, he was referred to our hospital by the local hospital. Upon hospital admission, the boy was noted to have atypical facial anomalies (Figure 3A). His physical examination revealed obvious developmental delay: his height was 122.0 cm [standard deviation (SD), - 3], his weight was 25.4 kg (SD, -2), and his head circumference was 49.8 cm (SD, -2). The remainder of his physical examination was unremarkable showing in Table 1 (23/10 to 23/11, 2019). After reviewing his medical history, a diagnosis of a high suspicion of HLH was made by the diagnostic criteria used in the HLH-2004 trial (Bergsten et al., 2017). After two chemotherapy cycles of corticosteroid, cyclosporin A and etoposide (VP16), his condition returned back to stable at the middle of November 2019, and bone marrow morphology was revaluated, showing acute lymphoblastic leukemia (ALL) with a proportion of leukemic blasts of 48% (reference intervals (RI): <5%) (Figure 3B). Continued chemotherapy and hematopoietic stem cell transplantation (HSCT) were recommended, but his parents refused. This boy was asked to abandon continued treatment and be taken home without further evaluation. One month later, death occurred at home owing to disease progression.

## Literature Review

Review of relevant literature revealed 45 cases with *CDK13*-related disorder to date (Hamilton and Suri, 2019; Yakubov et al., 2019), and genotype-phenotype correlations are not significant, despite of the potential generality in genotypes that 37 of all 45 affected individuals carry missense variants within the protein kinase domain (Figure 2A; Table 2) (Sifrim et al., 2016; Bostwick et al., 2017; Deciphering Developmental Disorders Study, 2017; Hamilton et al., 2018; Uehara et al., 2018; van den Akker et al., 2018; Hamilton and Suri, 2019; Yakubov et al., 2019). In 45 reported individuals with *CDK13*-related disorder, all variants have risen *de novo*, which hints they may not have the ability to reproduce. The median age of the reported cases is 8.3 years (age range, 0.5–54 years) with only three adults. This older adult appears to offer some assurance that his underlying genetic defect did not affect his long-term survival (van den Akker et al., 2018). In contrast, the case herein shows the prognosis concerning quality of life and longevity is not optimistic.

**TABLE 2:** Summary of major clinical findings of 45 already published individuals with *CDK13*-related disorder.

Clinical Finding	Literature		This Case
	Total n	Prevalence in Reported Cases n (%)	
Gender, male/female	45	15 (33.3)/30 (66.7)	Male
Age at review/(range), years	45	8.3/(0.5, 54.0)	9.0
*CDK13* variant, missense/others	45	37 (82.2)/8 (17.8)	Missense
Craniofacial dysmorphism	45	45 (100)	Yes
Feeding difficulties	39	28 (71.8)	No
Developmental delay	44	43 (97.7)	Yes
Intellectual disability	45	43 (97.7)	Yes
Autism spectrum disorder	29	12 (41.4)	Yes
Hypotonia	35	25 (71.4)	Yes
Epilepsy	30	7 (23.3)	No
Structural brain abnormality	28	13 (46.4)	Yes
Structural heart defect	43	17 (39.5)	No
Recurrent infections	9	5 (55.6)	No
Constipation	16	7 (43.8)	Yes
Cryptorchidism	—	—	Yes

Notably, five of nine reported cases present with immunological complications marked specifically by recurrent ear, upper respiratory tract, or gastrointestinal infections (Table 2), one with recurrent mouth ulcers and one with low levels of IgM and IgA (Hamilton et al., 2018). The current case does not present with remarkable infections during the first years of life, but at 9 years of age, he appears to present with recurrent infections in a half year period and more severe phenotypes—excessive immune activation—HLH. Indeed, it is possible that isolated low level of IgM is a secondary condition related to HLH or leukemia. Patients with immune disorders are at an increased risk of HLH and malignancies compared with a normal population (Shapiro, 2011; Bode et al., 2015), but to our knowledge, no malignant diseases have been reported in those with *CDK13*-related disorder.

## Discussion

Currently, ALL combination therapy typically results in cure rates of more than 90 percent in the developed world. However, in the developing world, patients with ALL continue to have relatively poor outcomes in part due to abandonment of treatment (Allemani et al., 2015; Cai et al., 2019), The reasons are multifaceted regarding this case forgoing needed health care. First of all, the underlying *CDK13*-related disorder is not diagnosed in time at his first onset. His ASD also poses difficulties in assessing response to treatment because of his deficits in social communication and the symptom of apathy more obvious after malignancy-associated HLH (M-HLH) onset. Moreover, he has already experienced a prolonged hospitalization and clinical deterioration, but also has received incorrect and irregular treatment for early-stage M-HLH, which may potentially lead to a poorer outcome. Unfortunately, due to his abandonment for continued treatment, we do not further evaluate additional prognostic indicators or determine whether he may benefit from more intensive therapy.

Overall prognosis is quite poor for any M-HLH despite of the favorable cure rates for childhood ALL. Despite clinical advances in recent decades, misdiagnosis of M-HLH remains a significant concern. Notably, HLH is often more immediately life-threatening than the malignancy itself (Lehmberg et al., 2015). Frequently in clinical practice, a diagnosis of HLH is made in the patient who partly meets the criteria. Prompt initiation of immunochemotherapy is essential for survival, although in many cases, corticosteroid monotherapy is sufficient (Jordan et al., 2011). However, this, in return, increases the risk of misdiagnosis because the underlying causes may be obscured.

It is possible that the leukemia was a coincidental finding in this boy with a *CDK13* gene variant. Besides, ALL could also be a secondary consequence of the extensive clinical deterioration of the patient and the previous incorrect treatment. However, only limited information is known about *CDK13*-related disorders, and further studies are necessary to determine whether those with *CDK13*-related disorders may share long-term survival with the absence of significant life-limiting sequelae or confer increased susceptibility to cancer development, and whether the cases with such specific conditions may benefit from gene therapy or HSCT.

Interestingly, several recent studies have described the connection of *CDK13* influencing cancer development, such as, ovarian cancer, prostate cancer, gastric cancers, hepatocellular carcinoma, etc. (Dong et al., 2018; Greenleaf, 2019; Qi et al., 2021; Tadesse et al., 2021; Wu et al., 2021). However, the role of *CDK13* in transcription remains poorly understood. *CDK13* is a complicated multi-tasking protein of central importance to proper gene expression and to genome stability, but it also probably plays yet undiscovered roles in other processes. Many studies have shown that *CDK12* and *CDK13* influence a large set of common processes, but they also influence a smaller set of distinct processes (Greenleaf, 2019). The data of a recent study fundamentally characterized both *CDK12* and *CDK13* as critical regulators of RNA processing (Fan et al., 2020). These findings have important implications in the context of tumorigenesis. We know that the loss of *CDK12* activity will lead to aberrant CTD (C-terminal repeat domain of RNA polymerase II) phosphorylation which can lead to ovarian and prostate cancers with unusual genome instabilities. In contrast to *CDK12*, few functional analyses of genetic aberrations in *CDK13* have been undertaken, and thus, their precise impact on cellular metabolism remains speculative (Hamilton and Suri, 2019). It is predicted that impaired function of *CDK13* protein would result in reduced phosphorylation of the CTD of RNA polymerase II (Greifenberg et al., 2016). Expression studies in the lymphoblastoid cells derived from patient with *CDK13*-related disorder suggested that the mutant transcript was subject to nonsense-mediated decay, hence would not produce a stable protein product (Hamilton et al., 2018; Hamilton and Suri, 2019). Therefore, a major goal of future work will be to determine what the *in vivo* activities and functions of *CDK13* actually are, and further detailed information may enable us to discern how its loss can lead to cancers.

However, there are certain limitations in the present study, such as without experimental verification, thus further studies are needed to elucidate the underlying mechanisms of the phenotypes described in individuals with *CDK13*-related disorder.

In conclusion, we report a case of a boy with CDK13-related disorder presenting with HLH prior to ALL. Immune disorders may represent previously unappreciated diagnostic clues to *CDK13*-related disorder, and should be highlighted and monitored in long-term follow-up of this newly identified syndrome. Probably leukemia was a coincidental finding in this boy, but the case herein suggests that individuals with *CDK13*-related disorder also face risk of developing cancers. Further investigation could be more informative to clarify this presentation because of limited information in affected cases.

## Data Availability

The original contributions presented in the study are included in the article/Supplementary Material, further inquiries can be directed to the corresponding authors.

## References

[B1] Allemani C. Weir H. K. Carreira H. Harewood R. Spika D. Wang X.-S. (2015). Global Surveillance of Cancer Survival 1995-2009: Analysis of Individual Data for 25 676 887 Patients from 279 Population-Based Registries in 67 Countries (CONCORD-2). Lancet 385, 977–1010. 10.1016/S0140-6736(14)62038-9 25467588 PMC4588097

[B2] Bergsten E. Horne A. Aricó M. Astigarraga I. Egeler R. M. Filipovich A. H. (2017). Confirmed Efficacy of Etoposide and Dexamethasone in HLH Treatment: Long-Term Results of the Cooperative HLH-2004 Study. Blood 130, 2728–2738. 10.1182/blood-2017-06-788349 28935695 PMC5785801

[B3] Bode S. F. Ammann S. Al-Herz W. Bataneant M. Dvorak C. C. Gehring S. (2015). The Syndrome of Hemophagocytic Lymphohistiocytosis in Primary Immunodeficiencies: Implications for Differential Diagnosis and Pathogenesis. Haematologica 100, 978–988. 10.3324/haematol.2014.121608 26022711 PMC4486233

[B4] Bostwick B. L. McLean S. McLean S. Posey J. E. Streff H. E. Gripp K. W. (2017). Phenotypic and Molecular Characterisation of CDK13-Related Congenital Heart Defects, Dysmorphic Facial Features and Intellectual Developmental Disorders. Genome Med. 9, 73. 10.1186/s13073-017-0463-8 28807008 PMC5557075

[B5] Cai J. Yu J. Zhu X. Hu S. Zhu Y. Jiang H. (2019). Treatment Abandonment in Childhood Acute Lymphoblastic Leukaemia in China: a Retrospective Cohort Study of the Chinese Children's Cancer Group. Arch. Dis. Child. 104, 522–529. 10.1136/archdischild-2018-316181 30705079

[B6] Deciphering Developmental Disorders Study (2017). Prevalence and Architecture of De Novo Mutations in Developmental Disorders. Nature 542, 433–438. 10.1038/nature21062 28135719 PMC6016744

[B7] Dong X. Chen G. Cai Z. Li Z. Qiu L. Xu H. (2018). CDK13 RNA Over-editing Mediated by ADAR1 Associates with Poor Prognosis of Hepatocellular Carcinoma Patients. Cell. Physiol. biochem. 47, 2602–2612. 10.1159/000491656 29996118

[B8] Even Y. Escande M.-L. Fayet C. Genevière A.-M. (2016). CDK13, a Kinase Involved in Pre-mRNA Splicing, Is a Component of the Perinucleolar Compartment. PLoS One 11, e0149184. 10.1371/journal.pone.0149184 26886422 PMC4757566

[B9] Fan Z. Devlin J. R. Hogg S. J. Doyle M. A. Harrison P. F. Todorovski I. (2020). CDK13 Cooperates with CDK12 to Control Global RNA Polymerase II Processivity. Sci. Adv. 6, eaaz5041. 10.1126/sciadv.aaz5041 32917631 PMC7190357

[B10] Greenleaf A. L. (2019). Human CDK12 and CDK13, Multi-Tasking CTD Kinases for the New Millenium. Transcription 10, 91–110. 10.1080/21541264.2018.1535211 30319007 PMC6602566

[B11] Greifenberg A. K. Hönig D. Pilarova K. Düster R. Bartholomeeusen K. Bösken C. A. (2016). Structural and Functional Analysis of the Cdk13/Cyclin K Complex. Cell Rep. 14, 320–331. 10.1016/j.celrep.2015.12.025 26748711

[B12] Hamilton M. J. Caswell R. C. Canham N. Cole T. Firth H. V. Foulds N. (2018). Heterozygous Mutations Affecting the Protein Kinase Domain of CDK13 Cause a Syndromic Form of Developmental Delay and Intellectual Disability. J. Med. Genet. 55, 28–38. 10.1136/jmedgenet-2017-104620 29021403 PMC5749303

[B13] Hamilton M. J. Suri M. (2019). CDK13-related Disorder. Adv. Genet. 103, 163–182. 10.1016/bs.adgen.2018.11.001 30904094

[B14] Jordan M. B. Allen C. E. Weitzman S. Filipovich A. H. McClain K. L. (2011). How I Treat Hemophagocytic Lymphohistiocytosis. Blood 118, 4041–4052. 10.1182/blood-2011-03-278127 21828139 PMC3204727

[B15] Lehmberg K. Sprekels B. Nichols K. E. Woessmann W. Müller I. Suttorp M. (2015). Malignancy-associated Haemophagocytic Lymphohistiocytosis in Children and Adolescents. Br. J. Haematol. 170, 539–549. 10.1111/bjh.13462 25940575

[B16] Qi J.-C. Yang Z. Lin T. Ma L. Wang Y.-X. Zhang Y. (2021). CDK13 Upregulation-Induced Formation of the Positive Feedback Loop Among circCDK13, miR-212-5p/miR-449a and E2F5 Contributes to Prostate Carcinogenesis. J. Exp. Clin. Cancer Res. 40, 2. 10.1186/s13046-020-01814-5 33390186 PMC7780414

[B17] Shapiro R. S. (2011). Malignancies in the Setting of Primary Immunodeficiency: Implications for Hematologists/oncologists. Am. J. Hematol. 86, 48–55. 10.1002/ajh.21903 21120868

[B18] Sifrim A. Hitz M. P. Hitz M.-P. Wilsdon A. Breckpot J. Turki S. H. A. (2016). Distinct Genetic Architectures for Syndromic and Nonsyndromic Congenital Heart Defects Identified by Exome Sequencing. Nat. Genet. 48, 1060–1065. 10.1038/ng.3627 27479907 PMC5988037

[B19] Tadesse S. Duckett D. R. Monastyrskyi A. (2021). The Promise and Current Status of CDK12/13 Inhibition for the Treatment of Cancer. Future Med. Chem. 13, 117–141. 10.4155/fmc-2020-0240 33295810

[B20] Uehara T. Takenouchi T. Kosaki R. Kurosawa K. Mizuno S. Kosaki K. (2018). Redefining the Phenotypic Spectrum of De Novo Heterozygous CDK13 Variants: Three Patients without Cardiac Defects. Eur. J. Med. Genet. 61, 243–247. 10.1016/j.ejmg.2017.12.004 29222009

[B21] van den Akker W. M. R. Brummelman I. Martis L. M. Timmermans R. N. Pfundt R. Kleefstra T. (2018). De Novo variants in CDK13 Associated with Syndromic ID/DD: Molecular and Clinical Delineation of 15 Individuals and a Further Review. Clin. Genet. 93, 1000–1007. 10.1111/cge.13225 29393965

[B22] Wang K. Li M. Hakonarson H. (2010). ANNOVAR: Functional Annotation of Genetic Variants from High-Throughput Sequencing Data. Nucleic Acids Res. 38, e164. 10.1093/nar/gkq603 20601685 PMC2938201

[B23] Wu Z. Wang M. Li F. Wang F. Jia J. Feng Z. (2021). CDK13-Mediated Cell Cycle Disorder Promotes Tumorigenesis of High HMGA2 Expression Gastric Cancer. Front. Mol. Biosci. 8, 707295. 10.3389/fmolb.2021.707295 34513922 PMC8427521

[B24] Yakubov R. Ayman A. Kremer A. K. van den Akker M. (2019). One-month-old Girl Presenting with Pseudohypoaldosteronism Leading to the Diagnosis of CDK13-Related Disorder: a Case Report and Review of the Literature. J. Med. Case Rep. 13, 386. 10.1186/s13256-019-2319-x 31883531 PMC6935476

