# Supplementary material for: Case Report: Hemophagocytic Lymphohistiocytosis Prior to the Onset of Leukemia in a Boy With CDK13-Related Disorder
Source: Front Genet. 2022 May 16;13:858668. doi: 10.3389/fgene.2022.858668 (PMC9149378; doi:10.3389/fgene.2022.858668)
Supplement: Supplementary file 1 [file Table1.DOCX]

**Supplementary Table. Full report of the variant found by NGS WES in the trio in the current case.**

| Gene | Location | Transcript; exon | Amino acids change | Homo/het | Allele frequency in population | Prediction | Variant classification | Modes of inheritance | Diseases/Phenotype | Parental origin |
| --- | --- | --- | --- | --- | --- | --- | --- | --- | --- | --- |
| *CDK13* | chr7:40039058 | NM_003718; exon4 | c.2141G>T (p.G714V) | het | - | D | Likely pathogenic | AD | Congenital heart disease, intellectual disability, developmental delay, and dysmorphic facial features | De novo |
| *BRWD3* | chrX:79980463 | NM_153252; exon15 | c.1490G>A (p.R497Q) | hemi | 0.0003 | B | Uncertain | XLR | X-linked mental retardation type 93 | Mother |
| *ABAT* | chr16:8862706 | NM_020686; exon11 | c.692A>G (p.K231R) | het | - | D | Uncertain | AR | GABA-transaminase deficiency | Mother |
| *AEBP1* | chr7:441535 20 | NM_001129; exon21 | c.3137C>G (p.P1046R) | het | - | B | Uncertain | AR | Ehlers-Danlos syndrome,classic-like, 2 | Father |
| *BCL3* | chr19:45262 680 | NM_005178; exon9 | c.1178-5C>T (splicing) | het | 0.0177 | - | Uncertain | - | Leukemia/B-cell lymphoma | Mother |
| *CACNA1B* | chr9:141016 333 | NM_000718; exon46 | c.6902C>A (p.T2301N) | het | - | B | Likely pathogenic | 1.AD 2.AR | 1.Dystonia type 23 2.Neurodevelopmental disorder with/without seizures | De novo |
| *CLC NKA* | chr1:163557 63 | NM_004070; exon12 | c.1196T>A (p.F399Y) | het | 0.0071 | B | Uncertain | DR | Bartter syndrome type 4B, biallelic status | Father |
| *CLC NKA* | chr1:163532 56 | NM_004070; exon7 | c.641C>G (p.A214G) | het | 0.0005 | B | Uncertain | DR | Bartter syndrome type 4B, biallelic status | Mother |
| *COL5A1* | chr9:137696 909 | NM_000093; exon40 | c.3203T>C (p.V1068A) | het | - | B | Uncertain | AD | Ehlers-Danlos syndrome, classical | Mother |
| *DNAH5* | chr5:139146 91 | NM_001369; exon10 | c.1258A>T (p.I420F) | het | - | B | Uncertain | AR | Primary ciliary dyskinesia type 3 | Father |
| *ENO3* | chr17:4859923 [1] | NM_053013; exon10 | c.1123G>A (p.E375K) | het | 0.0003 | D | Uncertain | AR | Glycogen storage disease type XIII | Mother |
| *EPHB4* | chr7:100411 302 | NM_004444; exon10 | c.1728C>G (p.D576E) | het | 0.0136 | B | Uncertain | 1.AD 2.AD | 1.{Edema, non-immune and/or atrial septal defect}  2.Capillary malformation-arteriovenous malformation type 2 | Father |
| *FAT2* | chr5:150924 968 | NM_001447; exon9 | c.5720T>C (p.I1907T) | het | 0.0053 | D | Uncertain | AD | Spinocerebellar ataxia type 45 | Father |
| *FOLR1* | chr11:71903 239 | NM_016725; exon2 | c.22C>A (p.Q8K) | het | 0.00004 | B | Uncertain | AR | Cerebral folic acid transport disorder neurodegenerative diseases | Father |
| *FXN* | chr9:71661314 [2] | NM_000144; exon2 | c.179G>A (p.R60H) | het | 0.0041 | B | Uncertain | AR | Friedreich ataxias | Father |
| *HPGD* | chr4:175439134-175439136 [3] | NM_000860; exon3 | c.310_311de l (p.L104Afs*3) | het | 0.0006 | - | Pathogenic | 1.AR 2.AR | 1.Congenital simple pestle finger 2.Primary hypertrophic osteoarthropathy type 1 | Mother |
| *HSPG2* | chr1:221988 03 | NM_005529; exon33 | c.4097G>A (p.R1366H) | het | 0.0009 | B | Uncertain | 1.AR 2.AR | 1.Silverman-Handmaker type dyssegmental dysplasia  2.Schwartz-Jampel syndrome type 1 | Father |
| *NDST1* | chr5:149907 642 | NM_001543; exon3 | c.790G>A (p.A264T) | het | 0.0012 | B | Uncertain | AR | Mental retardation type 46 | Mother |
| *JUP* | chr17:39913731 [4] | NM_002230; exon12 | c.1982G>A (p.R661Q) | het | 0.0025 | B | Uncertain | 1.AR 2.AD | 1.Naxos disease  2.Arrhythmogenic right ventricular cardiomyopathy/dysplasia type 12 | Father |
| *LAMB2* | chr3:491604 24 | NM_002292; exon27 | c.4286G>C (p.R1429P) | het | - | B | Uncertain | 1.AR 2.AR | 1.Pierson syndrome  2. Nephrotic syndrome type 5 | Mother |
| *LRP2* | chr2:170163815 [5] | NM_004525; exon4 | c.403G>A (p.D135N) | het | 0.0048 | D | Uncertain | AR | Donnai-Barrow syndrome | Mother |
| *MYL1* | chr2:211159 103 | NM_079420; exon4 | c.344C>G (p.P115R) | het | - | D | Uncertain | AR | Congenital myopathy with rapid convulsion ( type II ) fiber atrophy | Mother |
| *MYO5A* | chr15:52606 004 | NM_000259; exon41 | c.5457G>A (p.M1819I) | het | - | D | Uncertain | AR | Griscelli sydrome type 1 | Mother |
| *MYO5A* | chr15:52676 446 | NM_000259; exon15 | c.1826G>A (p.R609H) | het | 0.0001 | D | Uncertain | AR | Griscelli syndrome type 1 | Father |
| *OCA2* | chr15:28228553 [6] | NM_000275; exon14 | c.1441G>A (p.A481T) | het | 0.0303 | D | Uncertain | 1.AR 2.AR | 1.Albinism type II 2.Oculocutaneous albinism type 1 | Mother |
| *OGDH* | chr7:446849 51 | NM_002541; exon3 | c.248C>T (p.T83M) | het | 0.00001 | B | Uncertain | AR | α-ketoglutarate dehydrogenase deficiency | Mother |
| *PAK1* | chr11:77066 854 | NM_001128620; exon7 | c.631G>A (p.V211I) | het | - | B | Uncertain | AD | Mental development disorder with giant malformation seizures and speech retardation | Mother |
| *PCNT* | chr21:47852 070 | NM_006031; exon38 | c.8692A>G (p.R2898G) | het | - | B | Uncertain | AR | Microcephalic osteodysplastic primordial dwarfism type II | Father |
| *SLC2 6A4* | chr7:107342266 [7] | NM_000441; exon17 | c.1804-6G>A (splicing) | het | 0.0062 | - | Uncertain | 1.AR 2.AR | 1.Pendred syndrome  2.non-syndromic recessive deafness with an enlarged vestibular aqueduct, type 4 | Father |
| *PLEC* | chr8:144992422 | NM_201384; exon32 | c.11567G>A (p.S3856N) | het | - | B | Uncertain | 1.AR 2.AR 3.AR 4.AR 5.AD | 1. Limb-girdle muscular dystrophy 2Q  2.Epidermolysis bullosa simplex with muscular dystrophy 3.PLEC related epidermolysis bullosa with pyloric atresia 4.Epidermolysis bullosa simplex with nail dystrophy  5.Epidermolysis bullosa simplex type Ogna | Father |
| *PLOD1* | chr1:12014921 [8] | NM_000302; exon6 | c.614G>A (p.R205H) | het | 0.0006 | B | Uncertain | AR | Ehlers-Danlos symdrom type 6 | Father |
| *CTSA* | chr20:44519 984 | NM_000308; exon1 | c.20C>A (p.A7E) | het | 0.00008 | B | Uncertain | AR | Galactosialidosis | Mother |
| *PRK DC* | chr8:487467 53 | NM_006904; exon59 | c.8151+4A>G (splicing) | het | 0.0005 | - | Uncertain | AR | Immunodeficiency syndrome type 26 | Mother |
| *RYR1* | chr19:38974 035 | NM_000540; exon33 | c.4813A>T (p.S1605C) | het | - | D | Uncertain | 1.AR 2.AD, AR 3.AD | 1.Minicore myopathy  2.Central core disease  3.Malignant hyperthermia susceptibility type 1 | Mother |
| *SBF1* | chr22:50900 153 | NM_002972; exon22 | c.2644-6G>C (splicing) | het | 0.0108 | - | Uncertain | AR | Charcot-Marie-Tooth disease 4B3 | Father |
| *SLC6 A9* | chr1:444679 85 | NM_201649; exon8 | c.1181G>A (p.R394Q) | het | 0.00006 | D | Uncertain | AR | Glycine encephalopathy with normal glycine concentration | Father |
| *TFR2* | chr7:100230618 [9] | NM_003227; exon6 | c.849+6T>A (splicing) | het | 0.0186 | - | Uncertain | AR | Hereditary hemochromatosis type 3 | Father |
| *TFR2* | chr7:100228 539 | NM_003227; exon9 | c.1243G>A (p.G415S) | het | 0.0002 | B | Uncertain | AR | Hereditary hemochromatosis type 3 | Father |
| *TKT* | chr3:532646 27 | NM_001135055; exon8 | c.953G>A (p.R318H) | het | 0.00002 | D | Uncertain | AR | Short stature, congenital heart defects, and developmental delay | Mother |
| *TNXB* | chr6:320293 39 | NM_019105; exon21 | c.7327G>A (p.V2443M) | het | 0.00002 | B | Uncertain | 1.AR 2.AD | 1.Ehlers–Danlos Syndrome, Tenascin-X deficiency  2.Vesicoureteral reflux type 8 | Father |
| *TTN* | chr2:179410151 [10] | NM_133432; exon173 | c.68866G>A (p.E22956K) | het | 0.0013 | B | Uncertain | 1.AR 2.AD  3.-  4.AD 5.AR 6.AD | 1.Salih myopathy  2.Tibial muscular dystrophy 3.Dilated cardiomyopathy 1G 4.Familial dilated cardiomyopathy type 9  5.Limb-girdle muscular dystrophy type 10  6.Myofibromyopathy 9 with early respiratory failure | Father |
| *TTN* | chr2:179424 088 | NM_133432; exon155 | c.59951A>T (p.E19984V) | het | - | D | Uncertain | 1.AR 2.AD  3.-  4.AD 5.AR 6.AD | 1.Salih myopathy  2.Tibial muscular dystrophy 3.Dilated cardiomyopathy 1G 4.Familial dilated cardiomyopathy type 9  5.Limb-girdle muscular dystrophy type 10  6.Myofibromyopathy 9 with early respiratory failure | Father |
| *TTN* | chr2:179430 744 | NM_133432; exon155 | c.53295G>T (p.E17765D) | het | 0.0065 | B | Uncertain | 1.AR 2.AD  3.-  4.AD 5.AR 6.AD | 1.Salih myopathy  2.Tibial muscular dystrophy 3.Dilated cardiomyopathy 1G 4.Familial dilated cardiomyopathy type 9  5.Limb-girdle muscular dystrophy type 10  6.Myofibromyopathy 9 with early respiratory failure | Mother |
| *TTN* | chr2:179466 884 | NM_133432; exon113 | c.28301-7A>G (splicing) | het | - | - | Uncertain | 1.AR 2.AD  3.-  4.AD 5.AR 6.AD | 1.Salih myopathy  2.Tibial muscular dystrophy 3.Dilated cardiomyopathy 1G 4.Familial dilated cardiomyopathy type 9  5.Limb-girdle muscular dystrophy type 10  6.Myofibromyopathy 9 with early respiratory failure | Father |
| *TTN* | chr2:179529 587 | NM_001267550; exon166 | c.36110C>T (p.S12037L) | het | 0.0006 | B | Uncertain | 1.AR 2.AD  3.-  4.AD 5.AR 6.AD | 1.Salih myopathy  2.Tibial muscular dystrophy 3.Dilated cardiomyopathy 1G 4.Familial dilated cardiomyopathy type 9  5.Limb-girdle muscular dystrophy type 10  6.Myofibromyopathy 9 with early respiratory failure | Mother |
| *TTN* | chr2:179631 326 | NM_133432; exon40 | c.9347A>G (p.Q3116R) | het | 0.0048 | B | Uncertain | 1.AR 2.AD  3.-  4.AD 5.AR 6.AD | 1.Salih myopathy  2.Tibial muscular dystrophy 3.Dilated cardiomyopathy 1G 4.Familial dilated cardiomyopathy type 9  5.Limb-girdle muscular dystrophy type 10  6.Myofibromyopathy 9 with early respiratory failure | Mother |
| *VLDLR* | chr9:264557 1 | NM_003383; exon10 | c.1313-3C>T (splicing) | het | - | - | Uncertain | AR | Cerebellar ataxia, mental retardation and disequilibrium syndrome type 1 | Father |
| *XDH* | chr2:315967 56 | NM_000379; exon16 | c.1669G>A (p.D557N) | het | 0.013 | B | Uncertain | AR | Xanthinuria type I | Father |
| *PCGF2* | chr17:36891 805 | NM_007144; exon11 | c.706C>T (p.L236F) | het | 0.003 | B | Uncertain | AD | Turnpenny-Fry syndrome | Father |
| *ALMS1* | chr2:73677126 [11] | NM_015120; exon8 | c.3469G>A (p.A1157T) | het | 0.0001 | B | Uncertain | AR | Alstrom syndrome | Mother |
| *MOGS* | chr2:746900 80 | NM_006302; exon4 | c.836G>C (p.S279T) | het | 0.0012 | B | Uncertain | AR | Congenital Disorders of Glycosylation type Iib | Mother |
| *LHX3* | chr9:139089401 [12] | NM_014564; exon6 | c.979G>A (p.A327T) | het | 0.018 | B | Uncertain | AR | Combined pituitary hormone deficiency 3 | Mother |
| *LZTR1* | chr22:21348 261 | NM_006767; exon13 | c.1402C>T (p.R468C) | het | 0.0006 | B | Uncertain | 1.AD 2.AD 3.AR | 1.Neurofibromatosis type 2  2.Noonan syndrome type 10  3.Noonan syndrome type 2 | Mother |
| *TRRAP* | chr7:985011 44 | NM_003496; exon12 | c.1036+4C> T (splicing) | het | 0.0001 | - | Uncertain | AD | Developmental delay with or without autistic features | Mother |
| *MPDZ* | chr9:131685 64 | NM_003829; exon22 | c.3056-1G>T (splicing) | het | - | - | Likely pathogenic | AR | Non-syndromic hydrocephalus type 2 | Mother |
| *MPDZ* | chr9:131762 90 | NM_003829; exon20 | c.2776G>A (p.A926T) | het | 0.0034 | B | Uncertain | AR | Non-syndromic hydrocephalus type 2 | Father |
| *BSN* | chr3:496904 91 | NM_003458; exon5 | c.3502C>T (p.P1168S) | het | 0.00006 | B | Uncertain | - | Landau-Kleffner symdrom | Father |
| *RECQL4* | chr8:145738950 [13] | NM_004260; exon13 | c.2200+5G> A (splicing) | het | 0.00007 | - | Uncertain | 1.AR 2.AR 3.AR | 1.Rothmund-Thomson syndrome 2.Baller-Gerold syndrome 3.Rapadilino syndrome | Father |
| *LPIN2* | chr18:29252 72 | NM_014646; exon14 | c.1888G>A (p.G630S) | het | 0.0025 | B | Uncertain | AR | Majeed syndrome | Mother |
| *HDAC4* | chr2:240029 737 | NM_006037; exon15 | c.2097+9G>A (splicing) | het | 0.0018 | - | Uncertain | - | 2q37 microdeletions syndrome | Mother |
| *RUSC2* | chr9:355472 96 | NM_001135999; exon2 | c.778G>A (p.E260K) | het | 0.00001 | B | Uncertain | AR | Mental retardation type 61 | Mother |
| *KIF14* | chr1:200586 744 | NM_014875; exon2 | c.1108A>G (p.K370E) | het | 0.0027 | B | Uncertain | 1.AR 2.AR | 1.Meckel syndrome type 12 2.Primary microcephaly type 20 | Father |
| *HCN4* | chr15:73615363 [14] | NM_005477; exon8 | c.3071G>T (p.G1024V) | het | 0.0012 | B | Uncertain | 1.-  2.AD | 1.Brugada syndrome type 8  2.Sick sinus syndrome type 2 | Mother |
| *PIBF1* | chr13:73357 819 | NM_006346; exon2 | c.212C>T (p.T71I) | het | 0.0037 | B | Uncertain | AR | Joubert syndrome type 33 | Father |
| *DEAF1* | chr11:67874 2 | NM_021008; exon9 | c.1207A>G (p.I403V) | het | 0.0012 | B | Uncertain | 1.AD 2.AR | 1.Mental retardation type 24  2.Epilepsy, mental retardation or movement disorders | Father |
| *ARF GEF2* | chr20:47591 316 | NM_006420; exon13 | c.1679G>T (p.R560M) | het | 0.00006 | D | Uncertain | AR | Paraventricular gray matter heterotopia | Mother |
| *ADAMTS13* | chr9:136319 646 | NM_139025; exon24 | c.3154G>A (p.E1052K) | het | - | B | Uncertain | AR | Congenital thrombotic thrombocytopenic purpura | Mother |
| *MRPL3* | chr3:131188 550 | NM_007208; exon8 | c.806A>G (p.Y269C) | het | 0.0011 | B | Uncertain | AR | Complex-type oxidative-phosphorylation deficiency type 9 | Father |
| *ZNF423* | chr16:49672 014 | NM_015069; exon4 | c.1049C>T (p.S350F) | het | - | B | Uncertain | AD, AR | Nephronophthisis 14 | Mother |
| *KIAA0556* | chr16:27751 497 | NM_015202; exon15 | c.1879G>A (p.E627K) | het | 0.0105 | B | Uncertain | AR | Joubert syndrome type 26 | Father |
| *SYNE1* | chr6:152651 230 | NM_182961; exon78 | c.14590G>A (p.E4864K) | het | 0.00003 | B | Uncertain | 1.AD 2.AR 3.AR | 1.Emery-Dreifuss muscular dystrophy type 4  2.Arthrogryposis multiplex congenita, myogenic type  3.Autosomal recessive spinocerebellar ataxia type 8 | Mother |
| *EXOSC2* | chr9:133577 617 | NM_014285; exon7 | c.592G>A (p.G198S) | het | 0.00006 | D | Uncertain | AR | Short stature, hearing loss, pigmented retina and unique facial features | Mother |
| *TNNI 3K* | chr1:747971 40 | NM_015978; exon6 | c.451G>C (p.D151H) | het | 0.007 | B | Uncertain | AD | Cardiac conduction disease with or without dilated cardiomyopathy | Mother |
| *COQ4* | chr9:131087 455 | NM_016035; exon3 | c.236A>G (p.H79R) | het | - | B | Uncertain | AR | Primary coenzyme Q10 deficiency type 7 | Mother |
| *ANLN* | chr7:364501 75 | NM_018685; exon6 | c.1149A>C (p.E383D) | het | 0.0001 | B | Uncertain | AD | Focal segmental glomerulosclerosis type 8 | Father |
| *PRMT7* | chr16:68362 960 | NM_019023; exon6 | c.283-9A>G (splicing) | het | 0.0002 | - | Uncertain | AR | Short stature, short fingers, mental retardation and seizure | Mother |
| *MOCOS* | chr18:33800 117 | NM_017947; exon9 | c.1897C>T (p.R633W) | het | 0.0006 | B | Uncertain | AR | Xanthine urine type II | Mother |
| *MCCC1* | chr3:182738001 [15] | NM_020166; exon17 | c.1894C>T (p.P632S) | het | 0.002 | D | Uncertain | AR | 3-Methylcrotonyl coenzyme A carboxylase deficiency type 1 | Father |
| *HPSE2* | chr10:10050 3697 | NM_021828; exon4 | c.727A>T (p.S243C) | het | 0.0062 | B | Uncertain | AR | Urinary tract-face abnormality syndrome type 1 | Father |
| *MCCC2* | chr5:709485 86 | NM_022132; exon16 | c.1574+5G>A (splicing) | het | 0.0006 | - | Uncertain | AR | 3-Methylcrotonyl coenzyme A carboxylase deficiency type 2 | Mother |
| *SLC1 3A3* | chr20:45188833 [16] | NM_022829; exon13 | c.1637G>A (p.R546Q) | het | 0.0015 | B | Uncertain | AR | Acute reversible leukoencephalopathy accompanied by increased urinary ketoglutarate | Father |
| *WNK4* | chr17:40946 641 | NM_032387; exon13 | c.2305G>T (p.A769S) | het | - | B | Uncertain | AD | Pseudoaldosteronism type 2B | Mother |
| *CTC1* | chr17:81327 02 | NM_025099; exon19 | c.3074C>T (p.A1025V) | het | 0.0049 | D | Uncertain | AR | Cerebral retinal microvascular disease with calcification cyst | Mother |
| *ADGRV1* | chr5:899184 87 | NM_032119; exon5 | c.527G>A (p.G176E) | het | - | D | Uncertain | 1.AR 2.AD 3.AR | 1.Usher syndrome type IIC  2.Familial febrile seizures type 4  3.Usher syndrome type IIC (GPR98 / PDZD7 bigenotype ) | Mother |
| *GNPTG* | chr16:1412297 [17] | NM_032520; exon7 | c.502G>A (p.V168I) | het | 0.006 | B | Uncertain | AR | Viscous lipid accumulation syndrome type 3γ | Father |
| *PPP1R15B* | chr1:204378 672 | NM_032833; exon1 | c.1868T>C (p.V623A) | het | 0.0009 | B | Uncertain | AR | Microcephaly ,short limbs and glucose metabolism disorder type 2 | Father |
| *FRE M1* | chr9:148066 70 | NM_144966; exon19 | c.3263G>A (p.G1088D) | het | - | D | Uncertain | 1.AR 2.AR 3.AD | 1.Manitoba + Oculotrichoanal syndrome  2.Nasal fissure ( or with anorectal and renal malformations )  3.Trigonocephaly type 2 | Father |
| *TMPRSS6* | chr22:37466 517 | NM_153609; exon15 | c.1868+7G>A (splicing) | het | 0.00003 | - | Uncertain | AR | Iron-resistant iron deficiency anemia | Father |
| *PHACTR1* | chr6:130142 74 | NM_001322314; exon1 | c.156C>G (p.N52K) | het | 0.0136 | - | Uncertain | AD | Early infantile epileptic encephalopathy 70 | Father |
| *ASP M* | chr1:197074 035 | NM_018136; exon18 | c.4346T>C (p.L1449S) | het | 0.0002 | B | Uncertain | AR | Autosomal recessive primary microcephaly type 5 | Father |
| *MEFV* | chr16:3304626 [18] | NM_000243; exon2 | c.442G>C (p.E148Q) | het | 0.315 | B | Uncertain | 1.AD 2.AR | 1. Autosomal dominant familial Mediterranean fever  2. Familial Mediterranean fever | Father and Mother |
| *MEFV* | chr16:3299468 [19] | NM_000243; exon3 | c.1223G>A (p.R408Q) | het | 0.0641 | B | Uncertain | 1.AD 2.AR | 1. Autosomal dominant familial Mediterranean fever  2. Familial Mediterranean fever | Father |
| *MEFV* | chr16:3299586 [20] | NM_000243; exon3 | c.1105C>T (p.P369S) | het | 0.0884 | B | Uncertain | 1.AD 2.AR | 1. Autosomal dominant familial Mediterranean fever  2. Familial Mediterranean fever | Father |

Note：Prediction：Protein function prediction software REVEL (rare exome variant ensemble learners): D: deleterious; B: benign; - : Unknown

**References:**

[1] ClinVar:Uncertain significance;Glycogen storage disease type 13

[2] ClinVar:Likely benign(1);Uncertain significance(2);Cardiovascular phenotype

[3] HGMD: Osteoarthropathy, hypertrophic:Erken,et al.Mod Rheumatol,25,315,2015[PMI D:24533558]|24816859|25863089|28253600|29652982|30215240|30292630 ClinVar:Pathogenic;HPGD-Related Di sorders|Pachydermoperiostosis syndrome

[4] ClinVar:Uncertain significance;Naxos disease|Arrhythmogenic right ventricular cardiomyopathy; type 12

[5] ClinVar:Uncertain significance;Donnai Barrow syndrome

[6] HGMD: Albinism, oculocutaneous II:Lee,et al.N Engl J Med,330,529,1994[PMID:830 2318]|15942220|17568986|20981092|23324268|25333069|27468418

[7] HGMD: Hearing loss:Yang,et al.Hear Res,199,22,2005[PMID:15574297] ClinVar:Unc ertain significance;SLC26A4-Related Disorders

[8] ClinVar:Uncertain significance;Ehlers-Danlos syndrome; hydroxylysine-deficient

[9] ClinVar:Benign(1);Uncertain significance(1);Hereditary hemochromatosis

[10] ClinVar:Uncertain significance;-

[11] ClinVar:Uncertain significance;Alstrom syndrome

[12] ClinVar:Uncertain significance;-

[13] ClinVar:Uncertain significance;Baller-Gerold syndrome

[14] ClinVar:Uncertain significance;Brugada syndrome 8

[15] HGMD: 3-methylcrotonyl-CoA carboxylase deficiency:Shepard,et al.Genet Med,17,660,2015[PMID:25356967] ClinVar:Uncertain significance;3-MCC Deficiency|3 Methylcrotonyl-CoA carboxylase 1 deficiency

[16] ClinVar:Uncertain significance;Malignant tumor of prostate

[17] ClinVar:Uncertain significance;Mucolipidosis III Gamma

[18] HGMD: Mediterranean fever, familial, association with:Bernot,et al.Hum Mol Genet, 7,1317,1998[PMID:9668175]|10787449|11175300|11588211|12955725|14615741|15168590|15805719|17408446|19820229|19967574|20041150|20437121|20483145|20602240|20669279|20890251|21149248|21598804|21995303|22261745|22532615|22534884|22989844|23166428|23400211|23588594|23592051|23844200|23907647|23973724|24082139|24217079|24289199|24318677|24533551|24598070|24668705|24797171|24965843|25708585|25959027|26332735|26360812|26537665|27310525|27364639|27994174|28001092|28211254|28482392|28573371|28927886|2 9175894|29178647 ClinVar: Benign(1); Likely benign (4); Pathogenic(1); Uncertain significance(5); Familial Mediterr anean fever|Familial mediterranean fever; autosomal dominant

[19] HGMD: Mediterranean fever, familial:Cazeneuve,et al.Am J Hum Genet,65,88,1999 [PMID:10364520]|19934105|20041150|20525738|20981092|22906030|22975760|22995991|23291246|23400211|23524442|24082139|24797171|24965843|25708585|26360812|27513391|27884173|28001092|28927886|29178647 Cl inVar:Benign(1);Likely benign(1);Likely pathogenic(1);Uncertain significance(5);Familial Mediterranean fever

[20] HGMD: Mediterranean fever, familial:Aksentijevich,et al.Am J Hum Genet,64,949,19 99[PMID:10090880]|19934105|20041150|20981092|22906030|22975760|22995991|23291246|23400211|23524442|23907647|23981758|24082139|24797171|24965843|25708585|25959027|26027984|26360812|27535533|27884173| 28001092|28482392|28573371|28927886|29178647 ClinVar:Benign(1);Likely pathogenic(1);Uncertain significanc e(5);Familial Mediterranean fever
